# The complete mitochondrial genome of *Sardinella lemuru* (Clupeinae, Clupeidae, Clupeoidei) and phylogenetic studies of Clupeoidei

**DOI:** 10.1080/23802359.2017.1419085

**Published:** 2017-12-26

**Authors:** Hui Jiang, Li Gong, Liqin Liu, Bingjian Liu, Zhenming Lü

**Affiliations:** National Engineering Research Center of Marine Facilities Aquaculture, College of Marine Science and Technology, Zhejiang Ocean University, Zhoushan, People’s Republic of China

**Keywords:** *Sardinella lemuru*, mitogenome, phylogenetic relationship

## Abstract

The complete mitochondrial genome of *Sardinella lemuru* was sequenced using PCR amplification and primer walking sequence method. The complete mitochondrial genome of *S. lemuru* was 16,616 bp and contained 13 protein-coding genes, 22 transfer RNA genes, two ribosomal RNA genes, and one control region (D-loop). The overall base composition was A 25.04%, C 29.36%, G 20.40%, T 25.20%. In this study, the gene arrangement was consistent with other *Sardinella* mitochondrial genomes. Additionally, the phylogenetic relationships of 23 Clupeoidei species based on the complete genome was analyzed, and the result showed that *S. lemuru* firstly clustered with other two *Sardinella* species, *S. albella* and *S. maderensis.* These results would be useful for the investigation of phylogenetic relationship, taxonomic classification and phylogeography of the Clupeoidei.

*Sardinella lemuru*, belonging to Clupeinae, Clupeidae, Clupeoidei, Clupeiformes (Whitehead 1985; Nelson 2006), is an economically important species (Pedrosa-Gerasmio et al. 2015). It is distributed in the coastal water of China and the shallow sea of the western Pacific (Chen 2015). So far, the complete mitochondrial genome (mitogenome) sequence of this species has not yet been reported. In order to provide useful genetic information for genetic diversity, phylogenetic and taxology in future research, the complete mitochondrial genome of *S. lemuru* was determined (GenBank accession number MF536754).

*Sardinella lemuru* was collected from Zhejiang Province in the East China Sea (27°49'51”N; 121°04'25”E) with accession number 20160815SL26 and identified based on the morphologic features and COI gene. Total genomic DNA was extracted from muscle tissue of individual using the phenol-chloroform method (Barnett and Larson 2012). The universal primers (Ivanova et al. 2007) were designed according to the conserved regions of the complete mitogenome sequences of 26 Clupeoidei species downloaded from GenBank. Sequence alignment was conducted by BioEdit (Hall 1999). The phylogenetic tree involving 23 Clupeoidei species was constructed using the neighbor joining (NJ) methods based on the 13 protein-coding genes (Figure 1). The NJ trees were obtained with 10,000 bootstrap replications using MEGA5 (Tamura et al. 2011).

**Figure 1.**
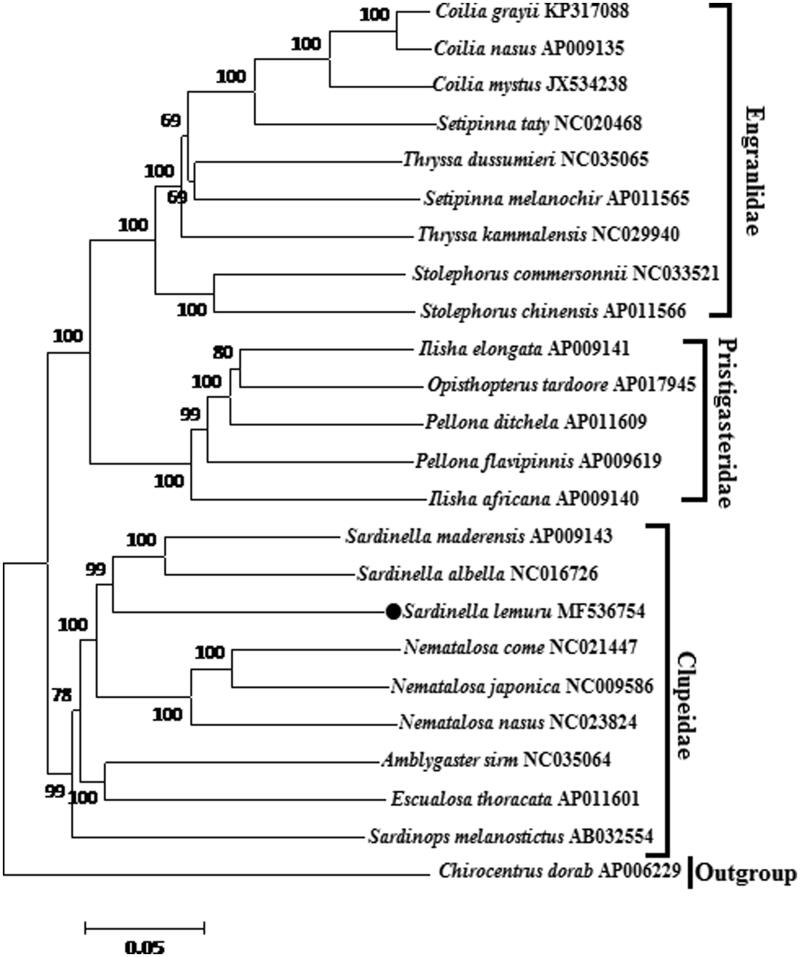
Neighbour-Joining tree of Clupeoidei based on the 13 protein-coding genes. The number at each node is the bootstrap probability. The number before the species name is the GenBank accession number. The dark spot indicates the species in this study.

Whole mitogenome sequence of *S. lemuru* was 16,616 bp in length, containing 13 protein-coding genes, 22 tRNA genes, two rRNA genes, and one control region. The gene arrangement and base content were similar with other teleosts (Shi et al. 2014; Yu and Kwak 2015; Gong et al. 2017). Almost all the protein-coding genes were encoded by H-strand with exception of *ND6* and eight tRNAs (Gln, Ala, Asn, Cys, Tyr, Ser, Glu, Pro) genes located on L-strand. The base composition was A 25.04%, C 29.36%, G 20.40%, T 25.20%. AT and GC contents were 50.24% and 49.76%, respectively, with the GC content (49.76%) higher than those found in most of the other Clupeoidei fishes (Bi and Chen 2011; Li, Shi, et al. 2012; Li, Zou, et al. 2012; Bo et al. 2013; Wang et al. 2016; Zhang et al. 2016). Twelve protein-coding genes started with an ATG initiation codon, while *COX1* used GTG as an initiation codon. For the termination codon, six protein-coding genes (*ND1*,* COX1*,* ATP8*,* ATP6*,* COX3*,* ND4L*) ended with TAA, four protein-coding genes (*ND2*,* ND3*,* ND5*,* ND6*) with TAG, and *COX2*,* ND4*,* CYTB* with T. The 13 protein-coding genes were 11,412 bp in length, accounting for 68.68% of the complete mitogenome, which encodes 3798 amino acids in total. The lengths of 12S rRNA located between tRNA^Phe^ and tRNA^Val^ and 16S rRNA located between tRNA^Val^ and tRNA^Leu^ were 957 bp and 1693 bp, respectively. The control region, located between tRNA^Pro^ and tRNA^Phe^, was 959 bp.

The phylogenetic tree of *S. lemuru* and other 22 Clupeoidei species was constructed using the neighbor joining (NJ) methods based on 13 protein-coding genes. The result showed that *S. lemuru* firstly clustered with other two *Sardinella* species, *S. albella* and *S. maderensis*, suggesting a very close relationship of these three species (Figure 1).
